# Total Hip Arthroplasty After Gunshot-Related Hip Injuries: Case Series and Review of Literature

**DOI:** 10.1016/j.artd.2025.101671

**Published:** 2025-03-26

**Authors:** Artsiom Abialevich, Ilan Tzaytlin, Asaf Acker, Vadim Benkovich

**Affiliations:** a Soroka University Medical Center, Be’er Sheva, Israel; b Ben-Gurion University of the Negev Faculty of Health Sciences, Be’er Sheva, Israel

**Keywords:** High velocity gunshot injury, Complex acetabular fractures, Staged hip arthroplasty, Multidisciplinary trauma management

## Abstract

High-velocity penetrating trauma to the hip can result in complex acetabular and femoral head fractures, often accompanied by vascular injuries and extensive soft tissue damage. Managing these injuries presents significant challenges due to contamination, bone loss, and the need for staged reconstruction. In 2 cases of severe ballistic and blast injuries, initial damage control measures included debridement, stabilization, and temporary antibiotic spacers to control infection risk. Delayed definitive total hip arthroplasty was performed after optimizing soft tissue and bony healing, leading to full functional recovery. This approach highlights the importance of staged reconstruction and multidisciplinary management in achieving successful outcomes in high-energy hip injuries.

## Introduction

Total hip arthroplasty (THA) is a well-established procedure for managing degenerative hip conditions, providing pain relief and restoring function [1]. However, high-velocity gunshot wounds (GSWs) to the hip pose unique challenges due to extensive bone and soft tissue damage, contamination, and associated vascular injuries. While GSWs account for approximately 2% of the 113,000 firearm-related injuries annually in the United States [2], their management remains complex, with limited literature guiding optimal treatment strategies [3,4]. The combination of infection risk, fracture patterns, and soft tissue compromise necessitates a multidisciplinary approach to achieve successful outcomes.

This article explores the management of GSW-related hip injuries, emphasizing a modified staged THA approach tailored to complex acetabular and femoral head fractures. By presenting 2 institutional case reports, we highlight the importance of initial damage control, infection prevention, and delayed definitive reconstruction. This approach provides insight into optimizing outcomes in high-energy penetrating hip trauma.

## Case history

### Initial presentation of case 1

**Figure 1:**
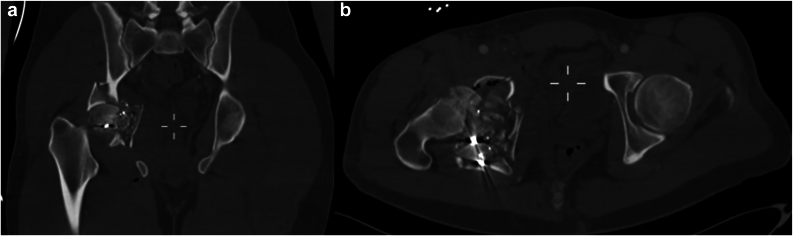
CT scan images in (a) coronal and (b) axial views of the right acetabulum and femoral head upon admission, showing severe comminution of the right proximal femur and acetabulum. Note the metal shrapnel from the high velocity projectile within the comminution (bright white dots). CT, computed tomography.

**Figure 2:**
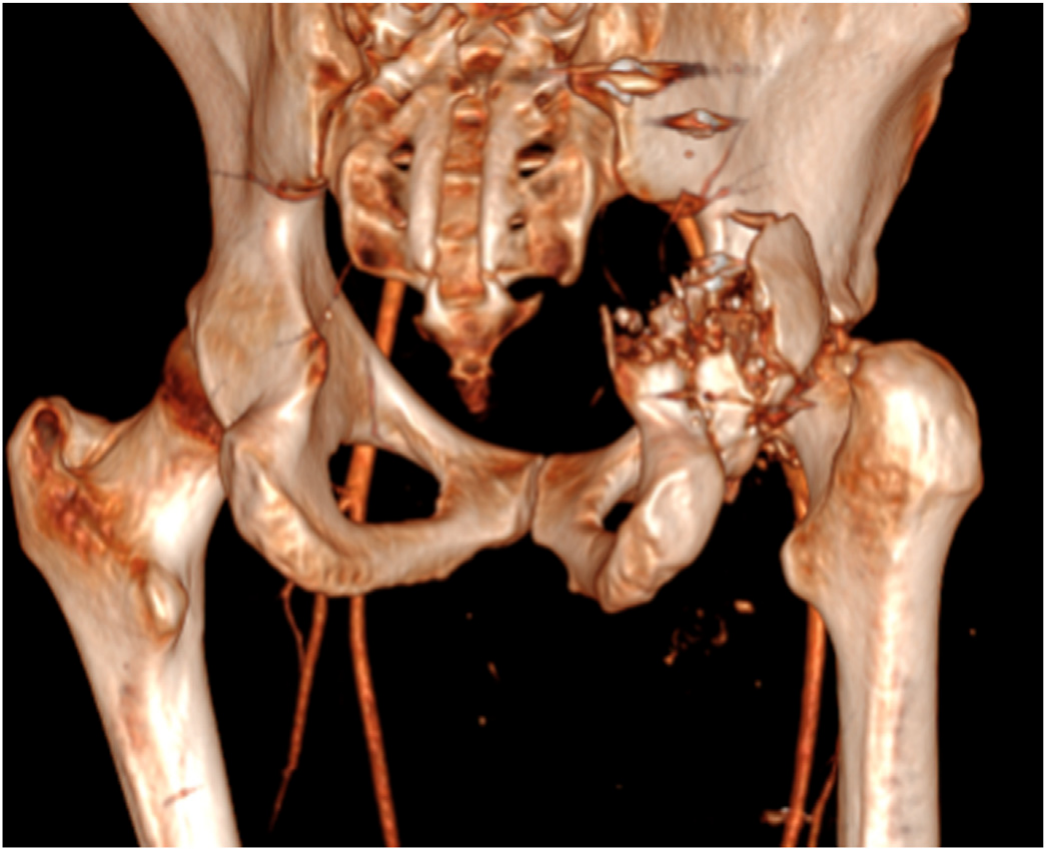
CT scan 3D reconstructive image of the right femur and acetabulum upon admission. Severe periarticular comminution is noted. 3D, 3-dimensional; CT, computed tomography.

**Figure 3:**
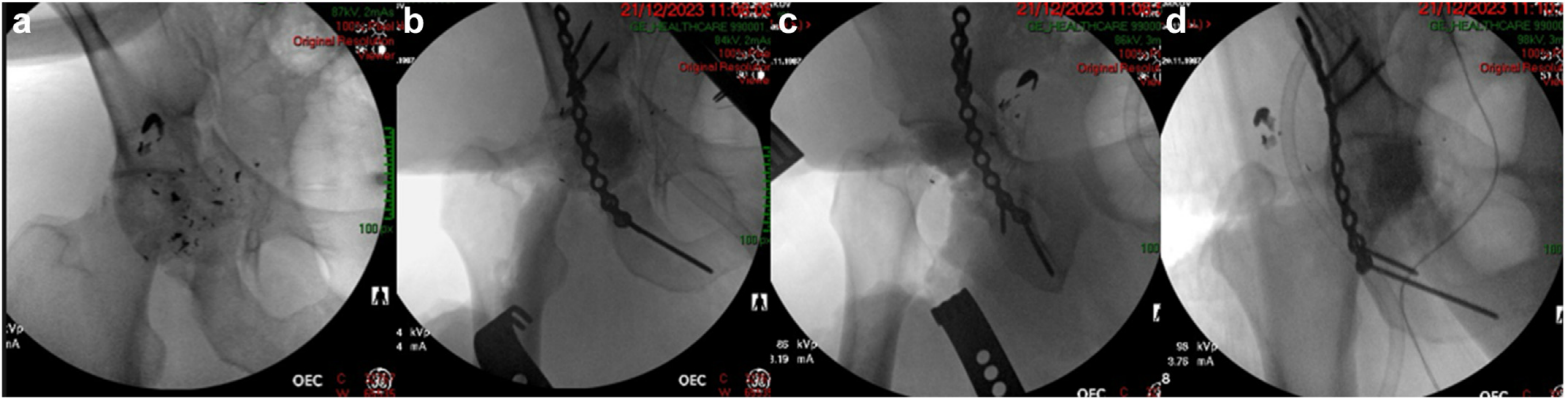
Intraoperative fluoroscopy demonstrating (a) prefracture and (b-d) postfracture stabilization of the right acetabulum, using screws and a reconstruction plate, with a cement spacer temporarily replacing the femoral head.

On December 14, 2023, a 36-year-old male was brought to the trauma bay following a GSW to the right groin. Upon evaluation, the patient was found to have open fractures of the right acetabulum and femoral head, along with a rupture of the femoral vein. Immediate surgical intervention was performed to explore and ligate the femoral vein, and to debride and irrigate the open fracture site. After stabilization in the intensive care unit, a multidisciplinary team was assembled to address the significant damage to the acetabular wall and joint stability, alongside the compromised femoral head (Figure 1, Figure 2). Given the patient’ s young age and the high risk of rapid osteoarthritis due to the severity of the injury, the primary surgical objectives were to achieve secure fixation that could serve as a scaffold for future joint arthroplasty, and to try and prevent infection (Fig. 3).

### Procedure

In the subsequent procedure, acetabular plate osteosynthesis was performed through the Kocher-Langenbeck approach. Fragments of the femoral head were excised to facilitate anatomical reduction of the acetabulum. An antibiotic spacer, molded from polymethyl methacrylate combined with 2 g of Vancomycin powder and 240 mg of Gentamycin, was constructed to maintain limb length, provide support for the acetabular fixation, and spread local high-dose antibiotics, as a preparation for the second stage of reconstruction.

Intraoperative cultures indicated the presence of Staphylococcus epidermidis, which was subsequently treated with intravenous Metronidazole and Cefuroxime. Three days after the surgery, the patient demonstrated an increase in inflammatory markers with purulent discharge from the groin wound and was taken to the operating room for another irrigation and debridement.

Cultures from this surgery revealed polymicrobial growth, including Pseudomonas, Klebsiella, Enterobacter spp., and Salmonella Group B, all of which were susceptible to Ciprofloxacin. The antibiotic regimen was adjusted accordingly based on these findings.

The initial management plan accounted for the elevated risk of infection commonly associated with GSWs, necessitating careful monitoring with imbedded potential for multiple interventions. Upon consultation from infectious diseases specialist, the intravenous antibiotic treatment spanned approximately 12 wks, with careful clinical and laboratory monitoring.

### Postoperation

Throughout the recovery process, the patient adhered to a nonweight-bearing protocol while attending regular follow-up appointments and engaging in physiotherapy to enhance hip range of motion. Since all inflammatory markers were normal, a decision was made not to aspirate the hip joint prior to the THA surgery, to lower the risk of causing a secondary infection in the process.

**Figure 4:**
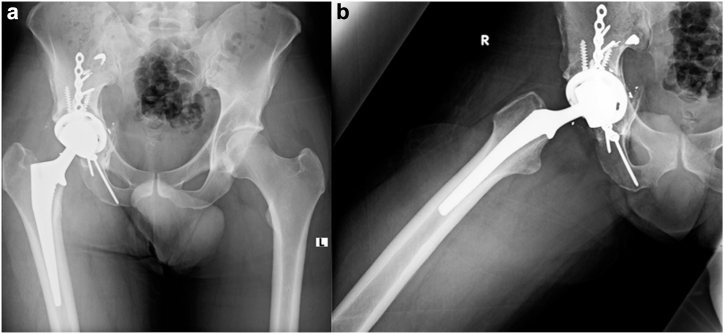
(a) AP and (b) axial radiographs taken at the 6-month follow-up of the right femur and acetabulum, demonstrating complete fracture healing and proper orientation of the THA components. AP, anteroposterior; THA, total hip arthroplasty.

Four months postinjury, the patient successfully underwent elective hip arthroplasty via the Kocher-Langenbeck approach (Fig. 4). Intraoperative findings revealed a well-healed pelvic construct, and a THA was performed, including a PINNACLE GRIPTION Multihole Acetabular Cup System DePuy Synthes secured with 3 screws and a high-offset CORAIL Total Hip System DePuy Synthes stem. At the end of the surgical procedure, a thorough washout was carried out using Saline + Iodine/Povidone solution with jet lavage, as per our regular arthroplasty protocol. No local antibiotics were used upon closure. Closure was facilitated by layers in a regular fashion—posterior capsule and short external rotators, and then fascia, subcutaneous, and skin. Cultures taken during surgery were sterile. Postsurgery, the patient was allowed full weight-bearing with cautions according to a limb lengthening protocol. Over the following weeks and months, he achieved significant functional improvement, regaining full physical health, including running and exercising with no restrictions or complaints impacting his daily activities. At the final follow-up, 1 year after the final operation, the patient demonstrated restored hip function and alignment, confirming the successful outcome of this complex case.

### Initial presentation of case 2

On October 7, 2023, a 44-year-old male was admitted following a traumatic injury sustained from a high velocity rifle and a hand grenade. Upon initial assessment, the patient presented with severe shrapnel and blast injuries, including a crushed frontal skull fracture with intraparenchymal shrapnel and pneumocephalus, a pneumothorax, an open fracture of the left acetabulum and femoral head and neck, and extensive soft tissue loss in the left upper arm.

### Procedure

**Figure 5:**
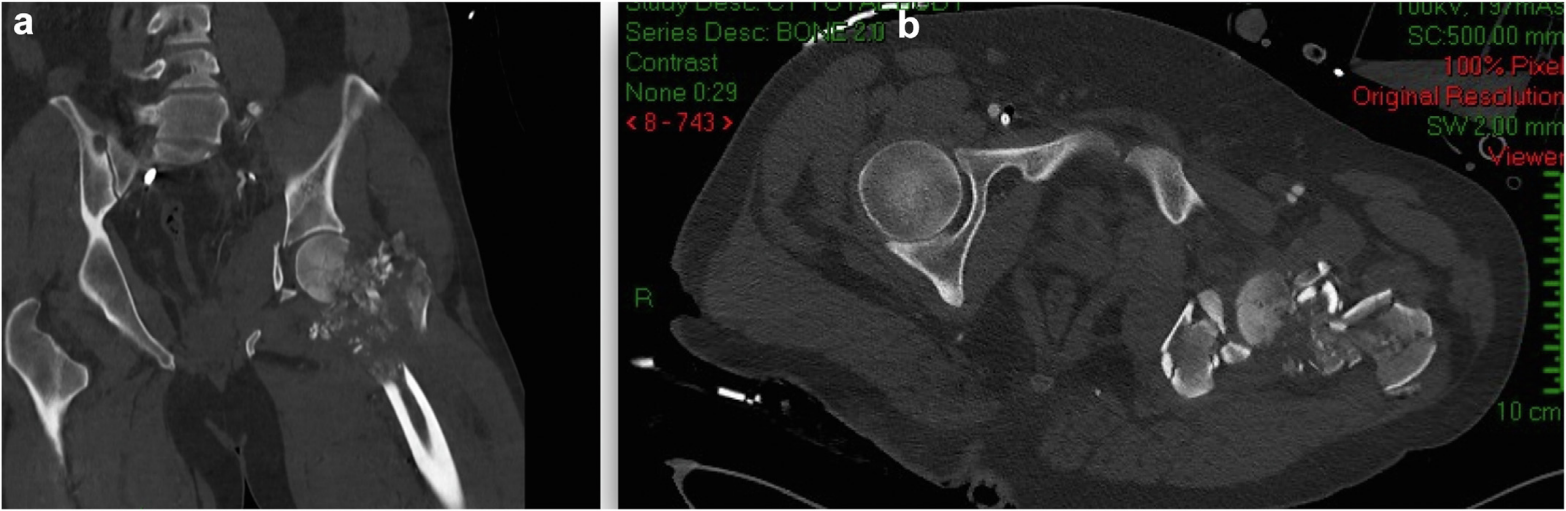
(a) Coronal and (b) axial CT scan cuts of the left femoral head and acetabulum upon admission. Severe periarticular comminution of the left proximal femur and left pelvic part is noted. CT, computed tomography.

**Figure 6:**
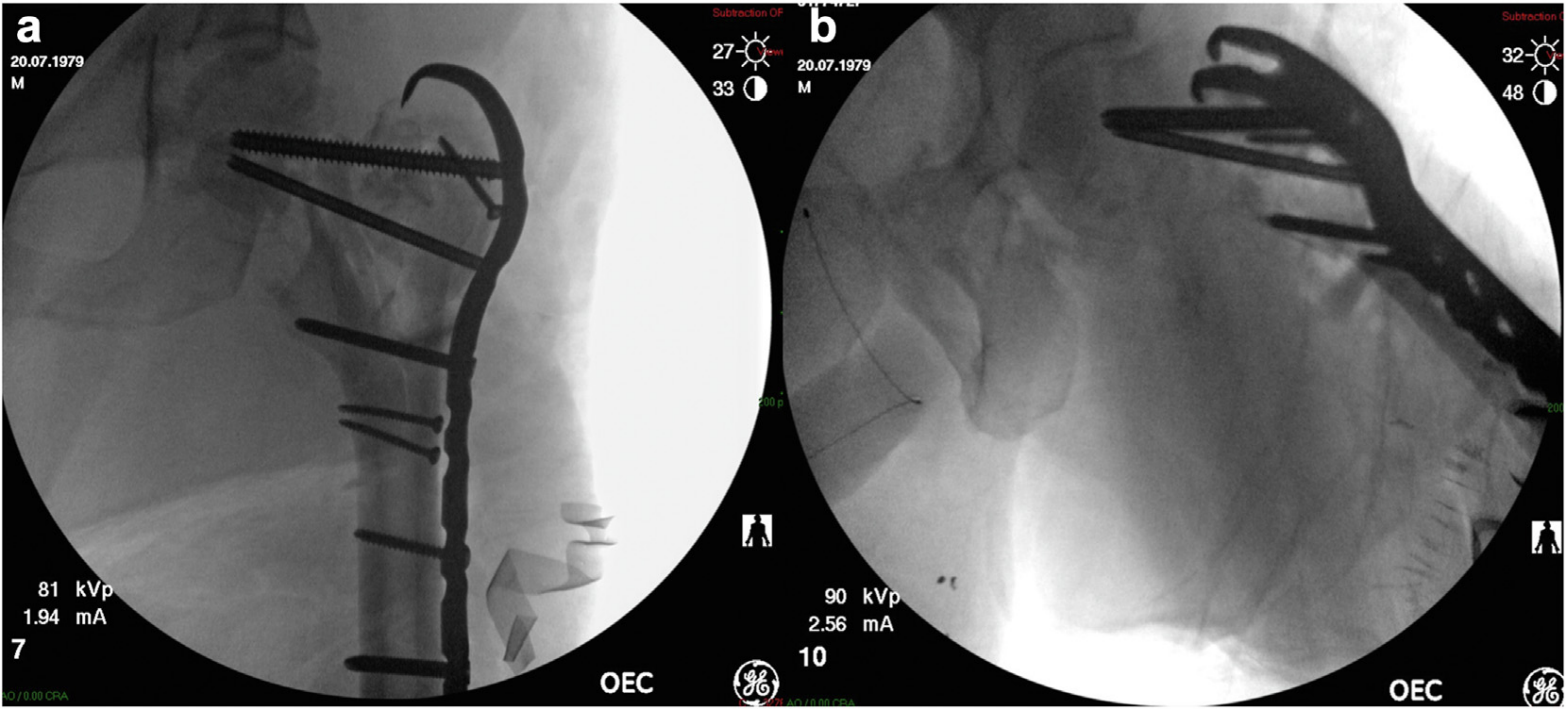
(a) AP and (b) axial views of intraoperative fluoroscopy demonstrating initial fracture stabilization of the left femur, using screws and a trochanteric hook plate. AP, anteroposterior.

The patient underwent urgent neurosurgical decompression to address the critical cranial injuries. Due to significant hemodynamic instability, only a quick irrigation and debridement was done to the left hip followed by skeletal traction through the left femur at that time, and the patient was subsequently transferred to the intensive care unit for close monitoring and further treatment. Four days later, surgical intervention was performed to address the orthopaedic injuries. The patient underwent irrigation and debridement of the soft tissue injuries, as well as irrigation of the open fractures of the acetabulum and femoral head. A comprehensive review of computed tomography scans (Fig. 5) and X-rays was conducted by the trauma unit team and the joint arthroplasty team. Due to the extensive comminution of the acetabulum and femoral head and neck and the significant risk of early post-traumatic osteoarthritis, the plan was to do a staged THA. The primary surgical focus was the reconstruction of the proximal femoral trochanteric part to build a reliable scaffold for the THA stem. A decision was made not to fix the acetabular fracture to lower the risk of subsequent infection, with the understanding that callus tissue will most likely form and create some medial support for the future THA cup. Subsequently, the patient underwent surgery via a lateral subvastus approach, wherein the entire proximal femur was reduced and stabilized using a 4.5 Hook Plate (Synthes) (Fig. 6).

Throughout the hospitalization, the patient received antibiotic prophylaxis targeting both his cranial and limb injuries. The initial regimen included Vancomycin and Piperacillin/Tazobactam, which was later adjusted to oral doxycycline based on clinical progress and laboratory cultures.

**Figure 7:**
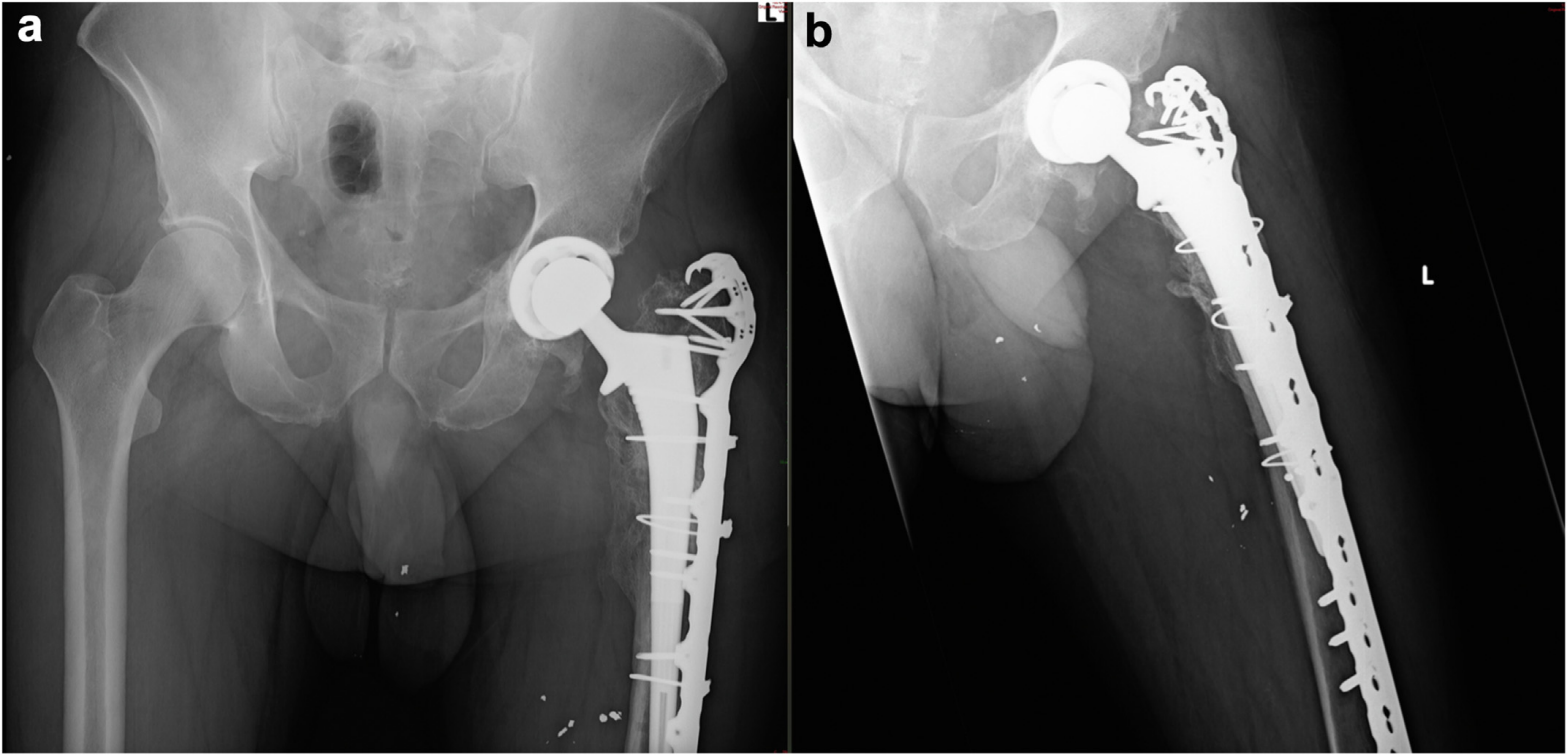
(a) AP and (b) axial X-ray views of the left proximal femur and hip joint at the 7-month follow-up, showing complete fracture healing and proper orientation of the THA components. AP, anteroposterior; THA, total hip arthroplasty.

Notably, all surgical and sanguineous cultures remained negative despite episodes of fever, which were later attributed to pulmonary complications. A joint aspiration was conducted a few weeks prior to the planned THA to ensure the absence of infection at the site. The results were sterile, allowing for the successful performance of THA 3 months postinjury (Fig. 7). The THA surgery was done through the postero-lateral approach, connected with the original lateral incision from the previous surgery. All the screws were initially removed from the plate and replaced with cables. A careful dissection was carried out in the postero-lateral area of the joint and the femoral head remanent was excised. The femoral canal was found to be almost completely blocked, and had to be opened using high-speed and manual drills. After the canal was ready, acetabular reaming was done, based on the healed medial acetabular wall, and a THA CORAIL Revision Stem DePuy Synthes with PINNACLE Acetabular Cup System DePuy Synthes and Ceramic-on-Polyethylene bearing was inserted. The final stage of the fixation was the reattachment of the plate to the bone with distal and para-stem screws. At the end of the surgical procedure, a thorough washout was carried out using Saline + Iodine/Povidone solution with jet lavage, as per our regular arthroplasty protocol. No local antibiotics were used upon closure. Closure was done by layers, starting with repair of the posterior capsule and the short external rotators, and then the fascia, subcutaneous, and skin. Cultures were not taken since the preoperative joint aspiration was sterile.

### Postoperation

Following surgery, the patient was guided through a rehabilitation program that included a nonweight-bearing protocol initially, with gradual progression every 2 wks to toe-touch, partial weight-bearing, and finally full weight-bearing as tolerated by 2 months. No other restrictions were applied. At the last follow-up, 7 months postsurgery, the patient demonstrated independent ambulation with a slight limp, full active and passive range of motion, and the ability to bear weight on the left leg. He regained full physical function and returned to his manual labor job. This successful rehabilitation exemplifies the effectiveness of our multidisciplinary approach to complex trauma management.

## Discussion

THA is a widely performed orthopaedic procedure. The hip’s complex anatomy and biomechanics make restoring its natural range of motion and mechanics after surgery a significant challenge [5].

The indications for THA have expanded from low-demand geriatric patients to encompass diverse pathologies. End-stage osteoarthritis remains the most common, with neoplastic involvement and postinfectious arthritis as less frequent but significant indications, reflecting THA’s evolving role in modern orthopaedics [6].

Subsequent to a gunshot injury, consequential soft tissue trauma, coupled with the prospect of neurovascular compromise, may accompany bone defects [7,8]. The removal of intra-articular bullets is crucial to prevent septic arthritis, lead toxicity, and cartilage damage. Additionally, the bullet’s trajectory through soft tissues can lead to complications such as infection, synovitis, cartilage erosion, foreign body reactions, and lead poisoning [[9], [10], [11]]. Thorough consideration and anticipation of these multifaceted concerns are indispensable in the judicious management of postgunshot injuries in hip joint [12].

### Pathophysiology of gunshot wounds to the hip

Gunshot injuries to the hip, caused by high-energy projectiles, damage soft tissues, bones, blood vessels, and pelvic structures, often resulting in fractures, joint instability, and functional limitations [13,14]. Complications such as infection, arthritis, and avascular necrosis frequently necessitate surgical intervention [14]. Veltre et al. [15] noted that comminuted fractures and bone loss from gunshot injuries complicate prosthetic fixation, while Obremskey et al. [16] emphasized the high infection risk due to wound contamination. Weiss et al. [17] highlighted post-traumatic arthritis as a common long-term complication, posing challenges for THA.

### Surgical techniques

Surgical management of post-traumatic arthritis after gunshot injuries is more complex than primary THA due to metallic fragments, soft tissue damage, and adhesions [18]. In our opinion, posterior approach offers superior joint visualization, aiding effective management in these challenging cases. Martin et al. [19] highlight the importance of careful planning for hip fixation, particularly in posterior wall acetabular fractures. The modified Gibson approach, with a straight lateral incision, provides single-access exposure, avoids gluteus maximus splitting, and preserves neurovascular structures, offering both functional and aesthetic advantages over the Kocher-Langenbeck approach. While open reduction internal fixation (ORIF) is the preferred technique for acetabular fractures in younger patients, no clear guidelines exist for the elderly. O'Toole et al. [20] reported a 25% 1-year mortality rate and a 28% conversion to arthroplasty within 2.5 years post-ORIF in patients aged more than 60 years, highlighting the challenges in this population. Current studies largely exclude primary fixation in civilian pelvic or hip gunshot fractures [21,22]. Recent studies suggest orthopaedic debridement is unnecessary for extra-articular pelvic fractures from low-velocity GSWs [[21], [22], [23], [24]]. However, for joint-penetrating injuries, most surgeons recommend routine hip arthrotomy to ensure thorough debridement, particularly in cases of bowel contamination, to prevent septic arthritis [3,25,26]. In acetabular fractures unrelated to GSWs, acute or delayed THA is an alternative but carries risks like deep vein thrombosis, aseptic loosening, heterotopic ossification, and infection [27,28]. For gunshot-induced hip injuries, Weinstein et al. [29] emphasize the high contamination of bullet remnants, increasing infection risk and guiding decisions on immediate vs delayed hip replacement.

### Clinical outcomes analysis

Ozden et al. [30] found THA to be an effective treatment for secondary hip arthritis caused by GSWs, improving patient outcomes. Bell et al. [31] documented a young patient with a comminuted femoral head and neck fracture from a gunshot, successfully treated with staged arthroplasty using an antibiotic spacer. Georgiadis et al. [32] reported satisfactory outcomes in a gunshot-induced femoral neck fracture treated with internal fixation and valgus osteotomy. Abdelaal et al. [33] found THA outcomes in patients with gunshot-induced secondary arthritis comparable to those for degenerative joint disease, indicating similar effectiveness. Pazarci et al. [3] demonstrated the feasibility of THA after debridement in young gunshot patients despite a moderate Harris score of 65.5%. Their study highlighted the challenges, including high infection rates, particularly with concurrent intestinal damage.

### Rehabilitation and postoperative care

In our opinion, the rehabilitation protocol following THA after high-velocity GSWs is generally analogous to that for patients recovering from periprosthetic fractures and those undergoing revision hip arthroplasty. In gunshot-related hip injuries, a delayed THA is often necessary to ensure infection is fully excluded, optimizing surgical outcomes [24]. For nongunshot injuries, Beaule et al. [34] suggest combining acute THA with ORIF in selected cases, offering earlier rehabilitation, reduced reoperations, and improved functional outcomes compared to ORIF alone or delayed THA. Rehabilitation protocols vary based on individual factors, requiring close collaboration among surgeons, rehabilitation specialists, and physical therapists.

### Complications and revision surgeries

Infection rates following THA for gunshot injuries are significant, with a 23% overall infection rate reported among 26 patients, including 14.3% for bullet injuries and 33.3% for shell fragment injuries [30]. While the literature lacks detailed data on hemorrhages, fracture complications, soft tissue damage, and nerve injuries, studies emphasize the complexity of managing these cases, focusing on infection control and functional outcomes. Vella et al. [35] highlighted the extensive physical, psychological, and social repercussions of GSWs, with survivors often experiencing poorer mental health and symptoms of post-traumatic stress disorder. This underscores the importance of comprehensive care addressing both physical recovery and psychological rehabilitation. The management of THA after gunshot injuries is more complex than standard procedures due to factors like foreign bodies, injury-specific challenges, and timing considerations. Meticulous surgical planning and execution are critical to minimizing complications and optimizing outcomes.

## Current controversies and future considerations

Future research trajectories in THA for GSW patients necessitate a multifaceted approach. We think that this includes the development of refined surgical techniques bespoke to the complexities of gunshot-related injuries, and the innovation of prosthetic designs to accommodate the specific anatomical alterations resultant from such trauma. Furthermore, the formulation of comprehensive rehabilitation programs, encompassing both physical and psychological rehabilitation, is imperative.

It is important to note that this report is based on 2 cases rather than a large clinical trial, limiting the generalizability of the findings. However, these cases provide valuable insight into the challenges and management strategies for high-velocity gunshot injuries to the hip, emphasizing the role of staged reconstruction in achieving successful outcomes.

## Summary

The existing literature on the treatment of hip fractures resulting from GSWs is inadequate. There are very few narrowly focused studies on the results of THA after GSWs.

There is an urgent need for high-quality randomized controlled trials to formulate reliable recommendations and guidelines. In our opinion, it is extremely important to conduct more high-quality studies on surgical debridement methods and the use of antibiotics for such fractures and the complications that arise subsequently.

This will facilitate the development of evidence-based guidelines that prioritize patient-centered care while ensuring cost-effectiveness.

**Key Points**

• Specialized surgical techniques tailored to the complexities of gunshot-related hip injuries may enhance outcomes.
• Prosthetic designs that address anatomical alterations resulting from high-velocity trauma could improve functional recovery.
• Comprehensive rehabilitation programs, incorporating physical and psychological components, are essential for holistic patient care.
• Current evidence is limited to case studies and small cohorts, necessitating cautious interpretation of findings.
• High-quality research on debridement techniques, antibiotic strategies, and long-term outcomes is critical for developing evidence-based guidelines.

## Informed patient consent

The author(s) confirm that written informed consent has been obtained from the involved patient(s) or if appropriate from the parent, guardian, power of attorney of the involved patient(s); and, they have given approval for this information to be published in this case report (series).

## Conflicts of interest

The authors declare there are no conflicts of interest.

For full disclosure statements refer to https://doi.org/10.1016/j.artd.2025.101671.

## CRediT authorship contribution statement

**Artsiom Abialevich:** Writing – review & editing, Writing – original draft, Data curation. **Ilan Tzaytlin:** Writing – review & editing, Data curation. **Asaf Acker:** Writing – review & editing, Supervision. **Vadim Benkovich:** Writing – review & editing, Conceptualization.

## Appendix A Supplementary data

Conflict of Interest Statement for Abialevich

Conflict of Interest Statement for Acker

Conflict of Interest Statement for Benkovich

Conflict of Interest Statement for Tzaytlin
