# Supplementary material for: Total Hip Arthroplasty After Gunshot-Related Hip Injuries: Case Series and Review of Literature
Source: Arthroplast Today. 2025 Mar 26;33:101671. doi: 10.1016/j.artd.2025.101671 (PMC11986253; doi:10.1016/j.artd.2025.101671)
Supplement: Conflict of Interest Statement for Abialevich [file mmc1.docx]

# CONFLICT OF INTEREST STATEMENT

***American Association of Hip and Knee Surgeons***

(Adopted from the American Academy of Orthopaedic Surgeons disclosure statement)

The following form **must be filled out completely and submitted by each author (example, 6 authors, 6 forms).**

**All items require a response. If there is no relevant disclosure for a given item, enter "*None*.”**

Title: Total Hip Arthroplasty After Gunshot-Related Hip Injuries: Case Series and Review of Literature

1. Royalties from a company or supplier (The following conflicts were disclosed) - none

2. Speakers bureau/paid presentations for a company or supplier (The following conflicts were disclosed) - none

3A. Paid employee for a company or supplier (The following conflicts were disclosed) - none

3B. Paid consultant for a company or supplier (The following conflicts were disclosed) - none

3C. Unpaid consultants for a company or supplier (The following conflicts were disclosed) - none

4. Stock or stock options in a company or supplier (The following conflicts were disclosed) - none

5. Research support from a company or supplier as a Principal Investigator (The following conflicts were disclosed) - none

6. Other financial or material support from a company or supplier (The following conflicts were disclosed) - none

7. Royalties, financial or material support from publishers (The following conflicts were disclosed) - none

8. Medical/Orthopaedic publications editorial/governing board (The following conflicts were disclosed) - none

9. Board member/committee appointments for a society (The following conflicts were disclosed) - none

**Each author must sign AND print or type his/her name, date and submit a separate form**

In addition, one BLINDED Conflict of Interest form (no author names used) should be submitted per manuscript with all
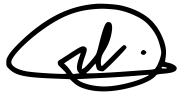
 author disclosures.

**Abialevich Artsiom** 10.12.2024

Author Name (Print or Type) Author Signature Date
